# Genetic Susceptibility to Astrovirus Diarrhea in Bangladeshi Infants

**DOI:** 10.1093/ofid/ofae045

**Published:** 2024-03-06

**Authors:** Laura Chen, Rebecca M Munday, Rashidul Haque, Dylan Duchen, Uma Nayak, Poonum Korpe, Alexander J Mentzer, Beth D Kirkpatrick, Genevieve L Wojcik, William A Petri, Priya Duggal

**Affiliations:** Department of Epidemiology, Johns Hopkins Bloomberg School of Public Health, Baltimore, Maryland, USA; Department of Genetic Medicine, Johns Hopkins University School of Medicine, Baltimore, Maryland, USA; Emerging Infections & Parasitology Laboratory, International Centre for Diarrhoeal Disease Research, Bangladesh, Dhaka, Bangladesh; Department of Epidemiology, Johns Hopkins Bloomberg School of Public Health, Baltimore, Maryland, USA; Center for Public Health Genomics, University of Virginia School of Medicine, Charlottesville, Virginia, USA; Department of Public Health Sciences, University of Virginia School of Medicine, Charlottesville, Virginia, USA; Department of Epidemiology, Johns Hopkins Bloomberg School of Public Health, Baltimore, Maryland, USA; The Wellcome Centre for Human Genetics, University of Oxford, Oxford, United Kingdom; Department of Microbiology and Molecular Genetics, Vaccine Testing Center, Larner College of Medicine, University of Vermont, Burlington, Vermont, USA; Department of Epidemiology, Johns Hopkins Bloomberg School of Public Health, Baltimore, Maryland, USA; Department of Medicine, Infectious Diseases and International Health, University of Virginia School of Medicine, Charlottesville, Virginia, USA; Department of Epidemiology, Johns Hopkins Bloomberg School of Public Health, Baltimore, Maryland, USA

**Keywords:** Astrovirus, diarrhea, host genetics, GWAS

## Abstract

**Background:**

Astroviral infections commonly cause acute nonbacterial gastroenteritis in children globally. However, these infections often go undiagnosed outside of research settings. There is no treatment available for astrovirus, and Astroviridae strain diversity presents a challenge to potential vaccine development.

**Methods:**

To address our hypothesis that host genetic risk factors are associated with astrovirus disease susceptibility, we performed a genome-wide association study of astrovirus infection in the first year of life from children enrolled in 2 Bangladeshi birth cohorts.

**Results:**

We identified a novel region on chromosome 1 near the loricrin gene (*LOR*) associated with astrovirus diarrheal infection (rs75437404; meta-analysis *P* = 8.82 × 10^−9^; A allele odds ratio, 2.71) and on chromosome 10 near the prolactin releasing hormone receptor gene (*PRLHR*) (rs75935441; meta-analysis *P* = 1.33 × 10^−8^; C allele odds ratio, 4.17). The prolactin-releasing peptide has been shown to influence feeding patterns and energy balance in mice. In addition, several single-nucleotide polymorphisms in the chromosome 1 locus have previously been associated with expression of innate immune system genes *PGLYRP4, S100A9,* and *S100A12*.

**Conclusions:**

This study identified 2 significant host genetic regions that may influence astrovirus diarrhea susceptibility and should be considered in further studies.

## Teaser

We identified 2 genetic polymorphisms associated with greatly increased risk of astrovirus diarrheal infections in the first year of life, the A allele of rs75437404 (odds ratio, 2.71) and the C allele of rs75935441 (odds ratio, 4.17).

## Graphical Abstract

**Figure:**
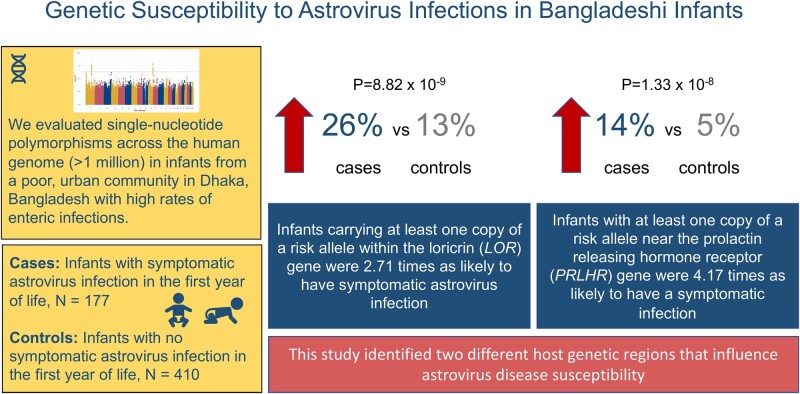

Diarrhea is the eighth leading cause of death across all ages and the second leading cause of death among children <5 years of age [1, 2]. There were 70.6 deaths per 100 000 children in this age group globally in 2016, compared with 1.3 deaths per 100 000 children within high-income countries [3]. Classic human astroviruses alone account for 2%–9% of all cases of acute nonbacterial gastroenteritis in children across the world, and an analysis of a Bangladeshi birth cohort found that 15%–20% of all diarrhea samples in the first year of life were positive for astrovirus [4, 5]. Astroviral infections are characterized by 2–3 days of watery diarrhea, but unlike many other enteric pathogens, astrovirus causes little histological change to intestinal epithelia, including inflammatory responses and cell death [4]. The virus can be recovered from the feces of asymptomatic children, suggesting the possibility of prolonged viral shedding or possibly a mechanism that allows astrovirus to remain in the gastrointestinal tract while causing epithelial barrier dysfunction [6–10].

Beyond gastrointestinal infections, there are reported cases of immunocompromised patients with encephalitis and meningitis, where astrovirus RNA has been detected in cerebrospinal fluid [11, 10]. Coupled with other known target organs in animals and the ability to infect across species, the burden of disease associated with astroviral infections may be higher than expected. In patients with gastroenteritis-associated astrovirus infections, the only available therapy is fluid replacement to avoid dehydration, as treatment for the virus does not exist.

Astrovirus is most commonly spread from person to person, especially via fecal-to-oral transmission and drinking water routes [12, 13]. There are 2 types of astrovirus, classic and novel, that are determined by genetic similarity; novel astroviruses are phylogenetically distant from classic human astroviruses [11]. Both classic and novel astroviruses circulate globally, with classic viruses more prevalent in developing countries [4, 10]. The persistence of astrovirus in high-income settings suggests that prevention of disease requires strategies beyond hygiene improvements [14]. Of the 3 open reading frames in the astrovirus genome, classic astroviruses share 64%–84% of capsid amino acid similarities and 93%–95% of nucleotide similarities in part of the second open reading frame [10]. Novel astroviruses belong to different clades than classic astroviruses, sharing up to 54% of amino acid identity with classic astroviruses [10]. The wide range of genetic diversity within Astroviridae makes potential vaccine development especially difficult.

We lack an understanding of the risk factors associated with astrovirus infections and disease severity. To identify host genetic risk factors associated with astrovirus disease susceptibility that may explain disease mechanism and vaccine targets, we performed genome-wide association studies in children enrolled in 2 birth cohorts, the Performance of Rotavirus and Oral Polio Vaccines in Developing Countries (PROVIDE) study and the Cryptosporidiosis and Enteropathogens in Bangladesh Birth Cohort (CBC) study in Dhaka, Bangladesh [15, 16]. We meta-analyzed the results and identified 2 novel regions on chromosomes 1 and 10 significantly associated with astrovirus diarrheal infections in infants.

## METHODS

The PROVIDE study protocol was approved by the Research Review Committee and Ethics Review Committee at the International Centre for Diarrhoeal Disease Research Bangladesh (icddr,b) and the institutional review boards of the University of Virginia and Vermont before implementation. The Ethics and Research Review Committees at icddr,b approved the CBC study. For both studies, informed written consent was obtained from the participants or the parents or guardians of all participants.

### PROVIDE Study Design

The PROVIDE study, including children from the Mirpur area of Dhaka, Bangladesh, aimed to evaluate the efficacy of oral and injectable vaccines using a randomized controlled clinical trial 2 × 2 factorial design [15]. From 2011 to 2016, the 700 children in the birth cohort and their mothers were followed up for the child's first 2 years of life, with biweekly diarrhea surveillance conducted in the homes by field research assistants. Active episodes of diarrhea were referred to the study clinic for evaluation and treatment. For each episode, a diarrhea stool specimen was collected. Height-for-age *z* (HAZ) and weight-for-age *z* (WAZ) scores were collected every 3 months.

### CBC Study Design

The CBC study investigated the disease burden of cryptosporidiosis and its effect on children's growth in urban and rural Bangladesh [16]. A total of 500 children from Mirpur, Dhaka, and 258 children from Mirzapur, a rural subdistrict located near Dhaka, were enrolled at birth. From 2014 to 2018, twice-weekly in-home visits were conducted by field research assistants to collect data regarding diarrhea and disease in children, and HAZ and WAZ scores were collected every 3 months. The study clinic was available to children and caregivers for development of symptoms of any illness. Stool samples were collected monthly and during episodes of diarrhea. Only a subset of children from the Mirpur site had polymerase chain reaction (PCR) testing of diarrheal samples (n = 220) and were therefore included in this study.

### Case and Control Definitions

Stool samples collected in both cohorts were tested for the presence of pathogens via real-time reverse-transcription PCR (RT-PCR) using TaqMan Array Cards [17]. Bar graphs displaying the distribution of RT-PCR cycle threshold (Ct) values for astrovirus in diarrhea samples were plotted using R v3.5.1 (Supplementary Figure 1). Case patients with diarrhea attributable to astrovirus were defined as children with diarrheal samples collected within the first year of life that resulted in RT-PCR Ct values for astrovirus >0 and <30. Children were defined as controls if they had ≥1 diarrhea sample available for testing from the first year of life but all RT-PCR Ct values for astrovirus were ≥30.

### Genotyping Array

In the PROVIDE study, children were genotyped on the Expanded Multi-Ethnic Genotyping Array (MEGA-EX) from Illumina, and those from the CBC study were genotyped on Illumina's Infinium Multiethnic Global Array (MEGA). These genetic data were phased with SHAPEIT software (version 2) and imputed with IMPUTE software (version 2.3.2) with 1000 Genomes Project phase 3 data as the reference. Standard quality control metrics were used for the genome-wide data. Single-nucleotide polymorphism (SNP) filters included genotype missingness <5%, minor allele frequency (MAF) >0.05, and Hardy-Weinberg equilibrium *P* > 10^−5^. The PROVIDE cohort had 10 792 283 initial variants, and the total genotyping rate was 0.99. A total of 590 340 variants were removed owing to missing genotype data, 1 487 431 were removed owing to minor allele threshold, and 131 were removed owing to Hardy-Weinberg exact test, leaving 8 777 081 variants.

In the CBC study, there were 10 942 212 initial variants and a total genotyping rate of 0.99. A total of 532 850 variants were lost owing to missing genotype data, 1 528 417 were removed owing to minor allele threshold, and 8 were removed owing to Hardy-Weinberg exact test, resulting in 8 880 118 variants that passed the quality control filters. Eighteen individuals in the CBC study were identified as outliers in the principal component analysis (PCA) based on their PCA score. Those with PCA scores falling 3 standard deviations above or below the median PCA value were removed (Supplementary Figure 2*A* and 2B). The genomic inflation factor, or λ, showed no inflation (λ=1.003 and 1.033 for PROVIDE and CBC studies, respectively) (Supplementary Figure 3).

### Association Analysis

Genome-wide association analyses [18] using homo sapiens (human) genome assembly GRCh37 (hg19) from the Genome Reference Consortium were performed separately for each study, using logistic regression with an additive model within SNPTEST version 2. Manhattan plots were constructed for each cohort using the ggplot2 package in R software, version 3.6.1, and highlights of regions of interest were created using LocusZoom tools at locuszoom.org with hg19/1000 Genomes South Asian as the reference genome build.

Data from the PROVIDE and CBC studies were combined for a fixed-effects meta-analysis using METAL software [19]. Input from both cohorts was filtered on MAF >5% and IMPUTE2 (INFO) score >0.7, retaining SNPs that had been imputed with high certainty in the data set. Functional Mapping and Annotation of Genome-Wide Association Studies (FUMA) was used to annotate genomic regions of interest and identify variants in linkage disequilibrium [20]. This annotation included expression quantitative trait loci (eQTLs) from several databases, including the Genotype-Tissue Expression project (GTEx) and the eQTLGen Consortium [21]. Conditional analyses were performed at each associated locus using SNPTEST version 2 in each cohort and then combined for an overall fixed-effects meta-analysis with METAL software.

## RESULTS

Within the PROVIDE study, 119 children were identified as having ≥1 astrovirus-associated diarrheal case in the first year of life, and 314 children did not have an associated astroviral infection. Children with astrovirus-associated diarrhea had a younger mean age (99.3 days) at the first diarrheal episode than children without astrovirus infection (123.6 days). Similarly, there were 58 case patients with astrovirus-associated diarrhea and 96 controls in the CBC study, with a mean age of first diarrheal episode of 117.7 days for case patients and 125.2 days for controls. In both cohorts, children not identified as case patients or controls either left the study early, did not have diarrhea, or had ≥1 sample with missing data for astrovirus. The distribution of HAZ and WAZ scores did not differ between case patients and controls in either cohort at birth or at 12 months of age (Table 1). Case patients and controls both had a mean mild diarrheal severity score in the PROVIDE study and a mean moderate diarrheal severity score in the CBC study (Table 1).

**Table 1.** Characteristics of Children in Performance of Rotavirus and Oral Polio Vaccines in Developing Countries (PROVIDE) and Cryptosporidiosis and Enteropathogens in Bangladesh Birth Cohort (CBC) Studies

Characteristic	Children in PROVIDE Study, No. (%)^a^		Children in CBC Study, No. (%)^a^	
	Case Patients (n = 119)	Controls (n = 314)	Case Patients (n = 58)	Controls (n = 96)

Age at first diarrheal infection, mean, d	99.3	123.6	117.7	125.2
Female sex	48 (40.3)	151 (48.1)	33 (56.9)	53 (55.2)
HAZ and WAZ scores at birth				
HAZ scores				
≤−2	13 (11.0)	31 (9.9)	7 (12.1)	12 (12.5)
−1.99 to ≤ 0	88 (74.0)	239 (76.1)	44 (75.9)	65 (67.7)
>0	18 (15.1)	44 (14.0)	7 (12.1)	19 (19.8)
WAZ scores				
≤−2	24 (20.2)	65 (20.7)	14 (24.1)	21 (21.9)
−1.99 to ≤ 0	85 (71.4)	231 (73.6)	38 (65.5)	71 (74.0)
>0	10 (8.4)	18 (5.7)	6 (10.3)	4 (4.2)
HAZ and WAZ scores at 12 mo				
HAZ scores				
≤−2	40 (33.9)	83 (27.0)	13 (22.4)	26 (27.1)
−1.99 to ≤ 0	67 (56.8)	203 (66.1)	42 (72.4)	60 (62.5)
>0	11 (9.3)	21 (6.8)	3 (5.2)	8 (8.3)
WAZ scores				
≤−2	33 (28.0)	68 (22.1)	9 (15.5)	19 (19.8)
−1.99 to ≤ 0	69 (58.5)	190 (61.9)	34 (58.6)	63 (65.6)
>0	16 (13.6)	49 (16.0)	15 (25.9)	12 (12.5)
Diarrheal severity: Ruuska score, mean	7.7	7.3	8.8	8.7
No. in household, mean	5.3	5.3	5.5	5.5
Principal source of household drinking water				
Municipality supply/piped water	114 (95.8)	305 (97.1)	58 (100)	95 (99.0)
Own arrangement by pump	3 (2.5)	9 (2.9)	0 (0)	1 (1.0)
Tube well	2 (1.7)	0 (0)	0 (0)	0 (0)
Principal type of toilet facility in household				
Septic tank or toilet	57 (47.9)	179 (57.0)	22 (37.9)	40 (41.7)
VIP with water seal	0 (0)	0 (0)	7 (12.1)	21 (21.9)
Water-sealed or slab latrine	52 (43.7)	121 (38.5)	27 (46.6)	34 (35.4)
Pit, open, or hanging latrine	10 (8.4)	14 (4.5)	2 (3.4)	1 (1.0)
Toilet facility shared with other households				
Yes	103 (86.6)	268 (85.4)	39 (67.2)	70 (72.9)
No	16 (13.4)	46 (14.6)	19 (32.8)	26 (27.1)
Household food availability				
Deficit all year	7 (5.9)	7 (2.2)	1 (1.7)	1 (1.0)
Deficit sometimes	31 (26.1)	109 (34.7)	1 (1.7)	1 (1.0)
Neither deficit nor surplus	55 (46.2)	132 (42.0)	11 (19.0)	14 (14.6)
Surplus	26 (21.8)	66 (21.0)	45 (77.6)	80 (83.3)

Abbreviations: CBC, Cryptosporidiosis and Enteropathogens in Bangladesh Birth Cohort; HAZ, height-for-age *z*; PROVIDE, Performance of Rotavirus and Oral Polio Vaccines in Developing Countries; VIP, ventilated improved pit; WAZ, weight-for-age *z*.

^a^Data represent no. (%) of children unless otherwise specified.

### Genetic Associations

**Figure 1.**
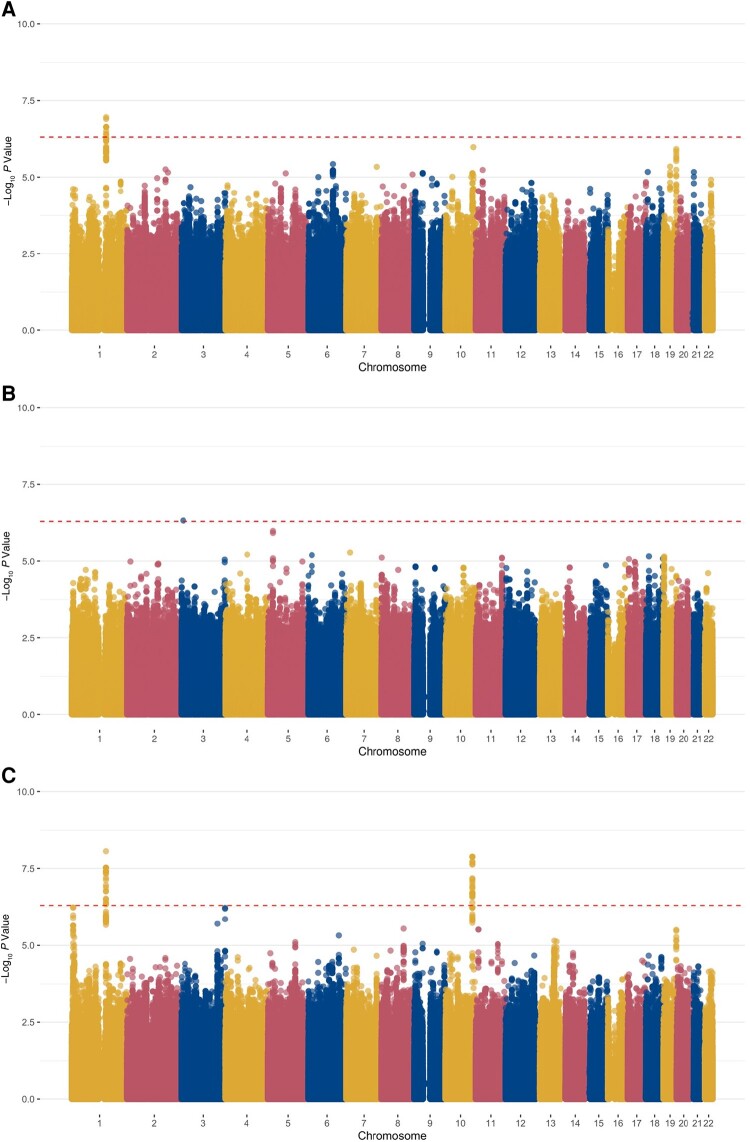
Single-nucleotide polymorphism (SNP) associations with astrovirus-associated diarrhea in the first year of life. Manhattan plots show −log_10_  *P* values for SNP associations by cohort, and dashed lines indicate thresholds for genome-wide significance (5 × 10^−7^). *A,* Performance of Rotavirus and Oral Polio Vaccines in Developing Countries (PROVIDE) study. *B,* Cryptosporidiosis and Enteropathogens in Bangladesh Birth Cohort (CBC) study *C,* Meta-analysis.

**Figure 2.**
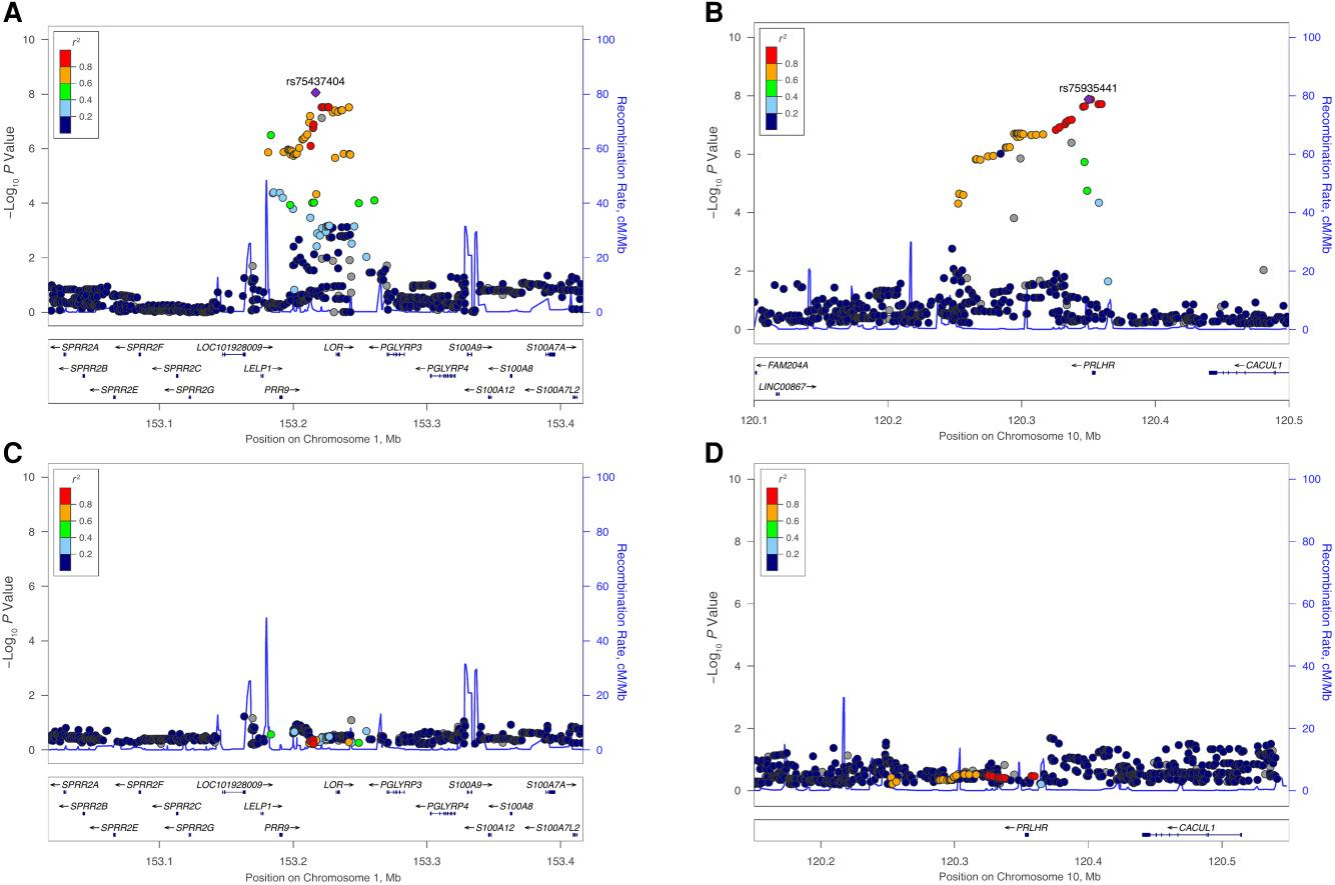
Regional association plots for 2 meta-analysis loci. *A*, Chromosome 1: *LOR* region. *B*, Chromosome 10: *PRLHR* region. *C*, Chromosome 1: *LOR* region conditioning on rs75437404. *D*, Chromosome 10: *PRLHR* region conditioning on rs75935441.

Figure 1 shows the association results for each cohort and the meta-analysis. The top association (rs75437404; meta-analysis *P* = 8.82 × 10^−9^; MAF, 16.7%) was identified in a noncoding region on chromosome 1 near the loricrin gene (*LOR*) (Table 2). The *LOR* gene is 2.4 kb in size, and the peak association region around this SNP spans 88 kb (Figure 2*A*) [22]. Children with ≥1 copy of the A allele at SNP rs75437404 were 2.7 times as likely to have an astrovirus-associated diarrheal infection within the first year of life as children with the T allele. This top associated SNP, rs75437404, has an MAF of 17.2% in the PROVIDE and 15.2% in the CBC study and is in linkage disequilibrium (*r*^2^ > 0.8), with 13 variants spanning an intergenic region 15.3 kb upstream of *LOR*. A conditional analysis incorporating rs75437404 in the model attenuated the association, suggesting that the region is in strong linkage disequilibrium and the alleles are correlated (Figure 2*C*).

**Table 2.** Genome-wide Significant Associated Single-Nucleotide Polymorphism From Stratified Analyses and Meta-analysis

SNP/Variant	Chromosome	Position	PROVIDE Study (n = 433)			CBC Study (n = 154)			Meta-analysis		
			OR	MAF	*P* Value	OR	MAF	*P* Value	OR	95% CI	*P* Value
rs75437404	1	153 216 817	2.60	0.17	1.09 × 10^−7^	2.12	0.15	1.81 × 10^−2^	2.71	1.93–3.81	8.82 × 10^−9^
rs12131158	1	153 221 315	2.53	0.19	2.28 × 10^−7^	1.94	0.17	2.99 × 10^−2^	2.47	1.79–3.40	3.02 × 10^−8^
rs7518709	1	153 225 883	2.53	0.19	2.28 × 10^−7^	1.94	0.17	2.99 × 10^−2^	2.47	1.79–3.40	3.02 × 10^−8^
rs7542150	1	153 225 921	2.53	0.19	2.27 × 10^−7^	1.94	0.17	2.99 × 10^−2^	2.47	1.79–3.40	3.01 × 10^−8^
rs6666892	1	153 226 579	2.53	0.19	2.28 × 10^−7^	1.94	0.17	2.99 × 10^−2^	2.47	1.79–3.40	3.02 × 10^−8^
rs6661601	1	153 233 510	2.39	0.20	6.22 × 10^−7^	2.02	0.17	1.89 × 10^−2^	2.44	1.77–3.36	4.46 × 10^−8^
rs12408942	1	153 241 677	2.44	0.20	3.95 × 10^−7^	2.01	0.17	1.96 × 10^−2^	2.47	1.79–3.41	3.06 × 10^−8^
rs114810342	10	120 346 096	2.91	0.07	1.60 × 10^−5^	4.82	0.07	2.80 × 10^−4^	4.05	2.48–6.62	2.38 × 10^−8^
rs55907016	10	120 347 235	2.91	0.07	1.54 × 10^−5^	4.81	0.07	3.96 × 10^−4^	4.06	2.48–6.63	2.30 × 10^−8^
rs75935441	10	120 350 104	2.99	0.07	7.71 × 10^−6^	4.58	0.07	3.96 × 10^−4^	4.17	2.55–6.83	1.33 × 10^−8^
rs116216801	10	120 350 154	2.99	0.07	7.71 × 10^−6^	4.58	0.07	3.96 × 10^−4^	4.17	2.55–6.83	1.33 × 10^−8^
rs115109981	10	120 350 780	2.99	0.07	7.70 × 10^−6^	4.57	0.07	3.71 × 10^−4^	4.17	2.55–6.83	1.34 × 10^−8^
rs117647802	10	120 351 875	2.99	0.07	7.69 × 10^−6^	4.57	0.07	3.72 × 10^−4^	4.17	2.55–6.82	1.34 × 10^−8^
rs116989176	10	120 357 518	3.00	0.08	7.43 × 10^−6^	4.22	0.07	6.19 × 10^−3^	4.08	2.50–6.66	1.95 × 10^−8^
rs1711891	10	120 359 829	3.00	0.08	7.42 × 10^−6^	4.23	0.07	6.18 × 10^−3^	4.07	2.50–6.65	1.93 × 10^−8^

Abbreviations: CBC, Cryptosporidiosis and Enteropathogens in Bangladesh Birth Cohort; CI, confidence interval; MAF, minor allele frequency; OR, odds ratio; PROVIDE, Performance of Rotavirus and Oral Polio Vaccines in Developing Countries; SNP, single-nucleotide polymorphism.

We also identified another region, including the prolactin releasing hormone receptor gene (*PRLHR*). The peak association region around *PRLHR* spans 51 kb, including the 9.9-kb gene (Figure 2*B*) on chromosome 10 (Figure 1*A*, 1*C*) [22]. SNP rs75935441 is in the 3' untranslated region of the *PRLHR* gene (meta-analysis *P* = 1.33 × 10^−8^; MAF, 7.5%) and is part of an enhancer sequence [23]. Children with ≥1 copy of the C allele at rs75935441 were 4 times as likely to have an astrovirus diarrheal infection as children with the T allele (odds ratio [OR], 4.17 [95% confidence interval, 2.55–6.83]). The conditional analysis including rs75935441 in the model attenuated the signal (Figure 2*D*).

**Figure 3.**
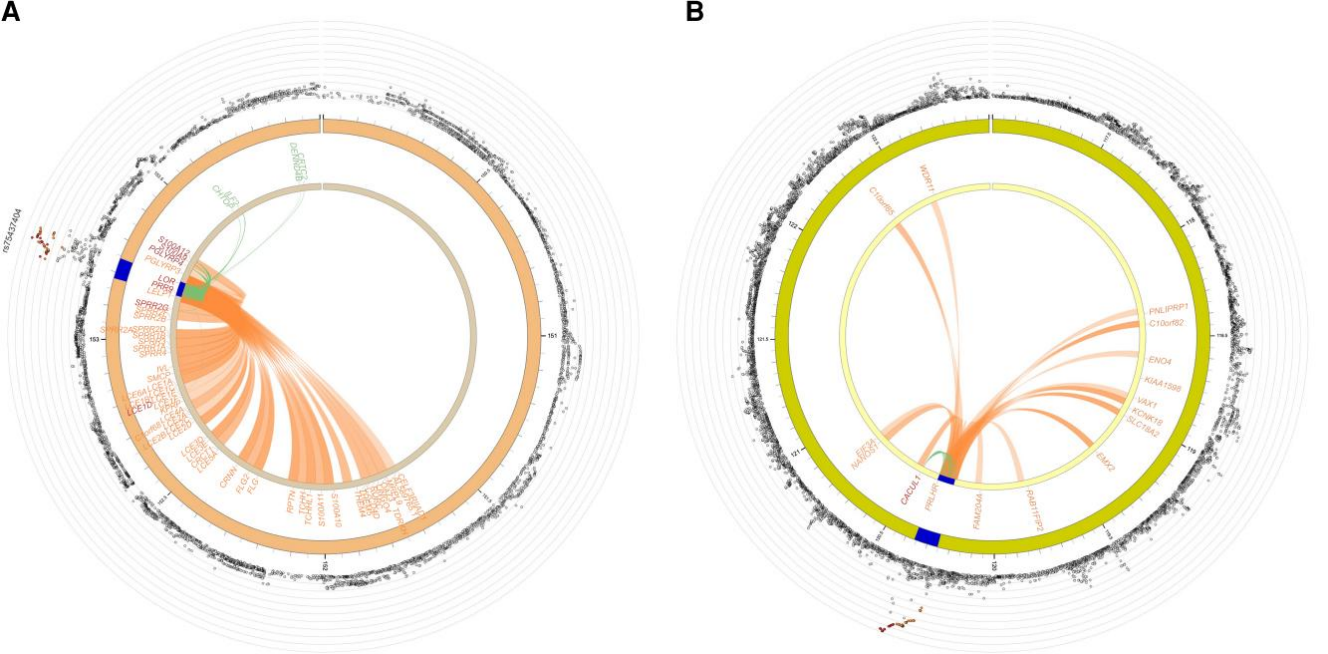
Circos plots of chromatin interactions. Outermost rings show results from the genome-wide association study meta-analysis for chromosomes 1 (*A*) and 10 (*B*). The next ring in each plot shows base pair coordinates along the chromosome (in megabases), with the region of interest colored blue. The innermost ring is annotated with gene symbols for all genes with a chromatin interaction (*orange*), an expression quantitative trait locus (*green*), or both (*red*).

Using FUMA, we identified chromatin interactions with the promoter regions of 55 genes on chromosome 1, including *LOR*, *PRR9*, *PGLYRP4*, *S100A9,* and *S100A12* (Figure 3*A* and Supplementary Table 1) spanning base pairs 153 181 362–153 242 630. Additionally, 11 genes had ≥1 eQTL in this region (Supplementary Table 2). Several variants have been previously associated with expression of multiple genes. For example, rs12125683 (C allele OR, 2.23, meta-analysis *P* = 7.92 × 10^−7^) is associated with expression of *PGLYRP4*, *S100A12*, and *S100A9*. The C allele of the SNP is associated with higher expression of *PGLYRP4* in esophagus mucosa (*P* = 2.69 × 10^−5^), as well as higher expression of *S100A12* (*P* = 1.94 × 10^−23^) and *S100A9* (*P* = 1.27 × 10^−6^) in whole blood (Supplementary Table 2).

Similarly, the chromosome 10 region (base pairs 120 252 606–120 359 829) had chromatin interactions with the promoter regions of 16 genes on chromosome 10, including *PRLHR*, *CACUL1*, *KCNK18*, *ENO4,* and *EIF3A* (Figure 3*B*, Supplementary Table 1). Only 1 gene also had identified eQTLs: *CACUL1*. The top associated SNP in this locus, rs75935441 (C allele OR, 4.17; meta-analysis *P* = 1.33 × 10^−8^), is an eQTL for *CACUL1,* and the C allele is associated with lower expression in both whole blood (*P* = 1.20 × 10^−12^) and skeletal muscle (*P* = 4.36 × 10^−6^) (Supplementary Table 2).

## DISCUSSION

We identified 2 significant regions across the genome that were associated with astrovirus diarrheal infection in the first year of life. The first region is upstream of the *LOR* gene and is associated with expression of immune system genes *PGLYRP4*, *S100A12*, and *S100A9*. The second region encompasses the *PLRHR* gene and is associated with expression of the gene *CACUL1*.

*LOR* encodes loricrin, which contributes to the protective barrier function of the epidermis and is expressed almost exclusively in mammalian stratified epithelia [24]. It is also found in macrophages in various tissues [25], but the relationship to astrovirus is not evident. However, within the chromosome 1 locus, the presence of chromatin interactions coupled with identification of eQTLs indicates that several of these variants are likely regulatory in nature. Both *S100A12* and *S100A9* are in the “MyD88-dependent cascade initiated on endosome” SuperPath [22]. Astrovirus enters cells via endocytosis, and when Toll-like receptors (TLRs) bind viral nucleic acid inside the cell, they initiate signaling cascades which include MyD88 and type 1 interferon [26, 27]. The S100A9 protein regulates TLR3 activation [28, 29], and the S100A12 protein is an endogenous activator of TLR4 [30]. Both proteins are calgranulins and in addition to TLR activation are also involved in protecting the body against damage from inflammation [31, 32]. The same allele associated with higher expression of these genes in whole blood is associated with increased odds of astrovirus-positive diarrhea in the first year of life. The C allele of rs12125683 is also associated with higher expression of *PGLYRP4* in esophagus mucosa. *PGLYRP4* is a peptidoglycan recognition protein, involved in the innate immune response to bacteria [22]. Higher expression of this gene could contribute to astrovirus susceptibility via changes to the gastrointestinal microbiome [33].

The *PRLHR* gene is also in a region significantly associated with astrovirus diarrheal infections. This gene encodes the receptor for prolactin-releasing peptide, which has been identified as a target for obesity treatment [34]. In mouse and rat models, prolactin-releasing peptide influences feeding patterns and energy balance, and it has shown diet-suppressing effects [34, 35]. However, we further stratified by WAZ and HAZ scores to account for any underlying malnutrition at birth or at 12 months of age that may predispose an individual to an infection, and there were no differences between case patients and controls (Table 1). Thus, the association between *PRLHR* and astrovirus diarrhea infections is not likely to be mediated by malnutrition. Further investigation to identify the potential association between *PRLHR* and the astrovirus replication cycle or its pathogenic mechanism is warranted.

In addition to the effects on *PRLHR*, we found SNPs associated with increased risk of astrovirus-positive diarrhea that were also associated with lower expression of the gene *CACUL1* in whole blood. This gene promotes cell proliferation and higher expression has been linked to increased invasion of gastric cancer cells [36]. While this gene is linked to *Helicobacter pylori* activity [36], it is unclear how it may be involved in astrovirus pathogenesis. Additional chromatin interactions at this locus suggest the potential for regulation of the genes *ENO4*, *KCNK18*, and *EIF3A*, but there are not yet data showing differences in gene expression based on genotypes found in the region of interest.

In conclusion, we identified 2 genetic regions associated with astrovirus infection susceptibility. Both regions warrant additional exploration for their potential association with immune and gastric cells and may elucidate astrovirus infection pathways.

## Supplementary Material

ofae045_Supplementary_Data

## Supplementary Data

Supplementary materials are available at *Open Forum Infectious Diseases* online. Consisting of data provided by the authors to benefit the reader, the posted materials are not copyedited and are the sole responsibility of the authors, so questions or comments should be addressed to the corresponding author.
