# Supplementary material for: Genetic Susceptibility to Astrovirus Diarrhea in Bangladeshi Infants
Source: Open Forum Infect Dis. 2024 Mar 6;11(3):ofae045. doi: 10.1093/ofid/ofae045 (PMC10960603; doi:10.1093/ofid/ofae045)
Supplement: ofae045_Supplementary_Data [file ofae045_supplementary_data.zip › SuppFigure1.docx]

**a.**


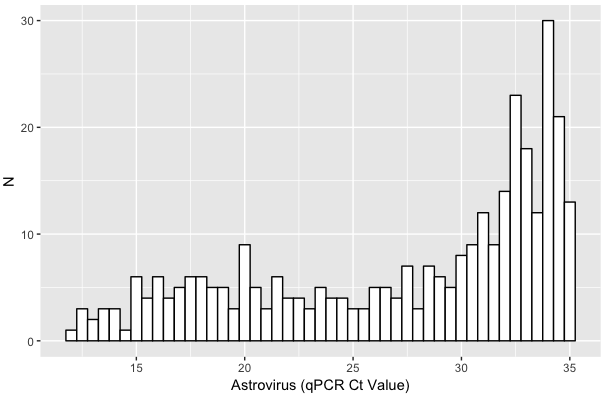


**b.**

**
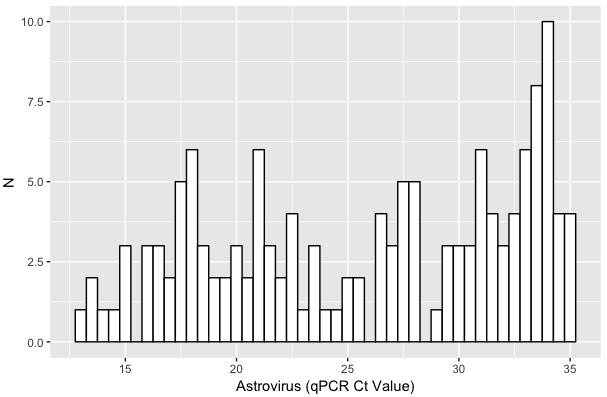
**

# **Supplementary Figure 1.** Distribution of astroviral load among diarrheal events by cohort. Bar graphs showing frequency of each Ct value, produced in R v3.5.1. **a)** PROVIDE. **b)** CBC.
