# Supplementary material for: Genetic Susceptibility to Astrovirus Diarrhea in Bangladeshi Infants
Source: Open Forum Infect Dis. 2024 Mar 6;11(3):ofae045. doi: 10.1093/ofid/ofae045 (PMC10960603; doi:10.1093/ofid/ofae045)
Supplement: ofae045_Supplementary_Data [file ofae045_supplementary_data.zip › SuppFigure2.docx]

**a)**


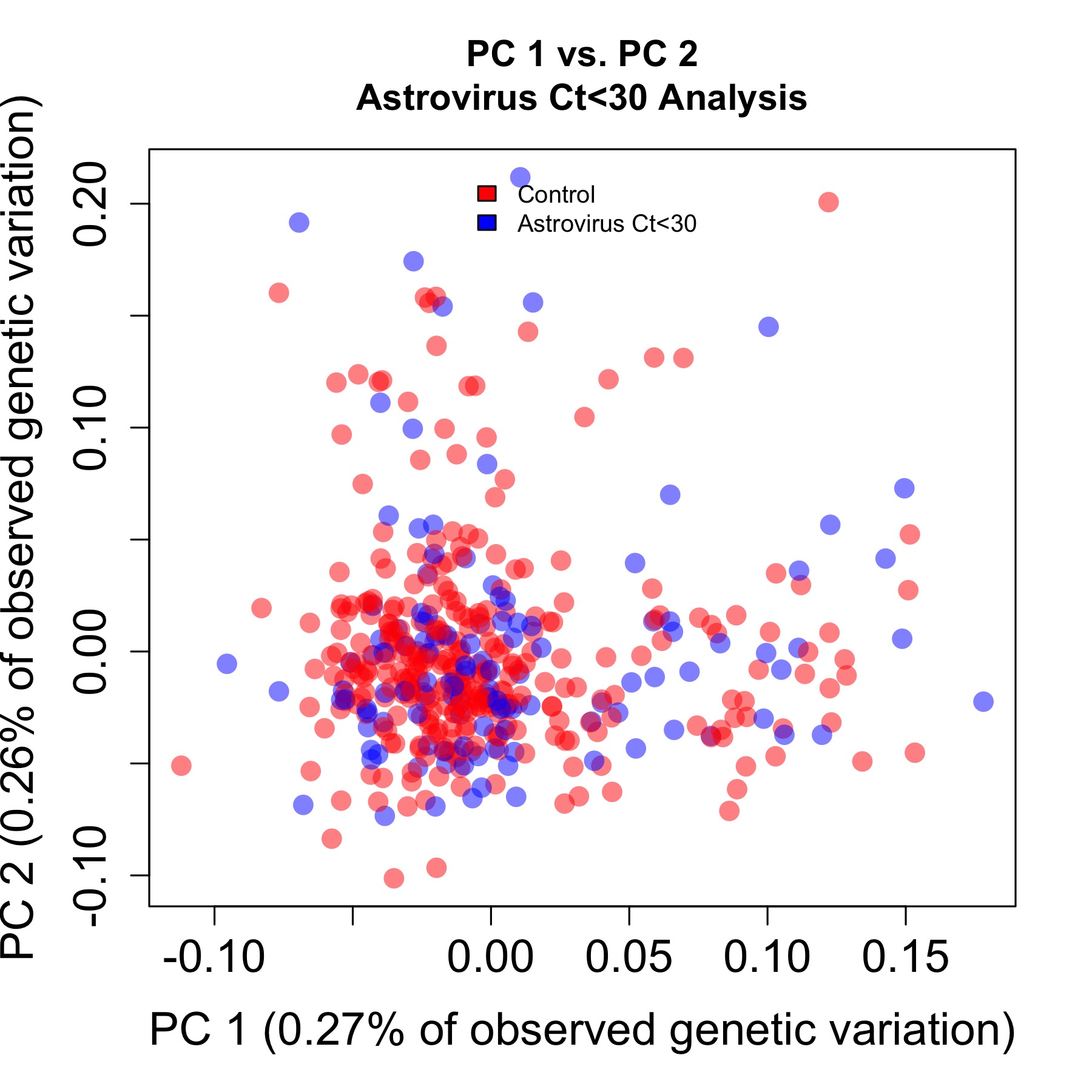


**b)**

**
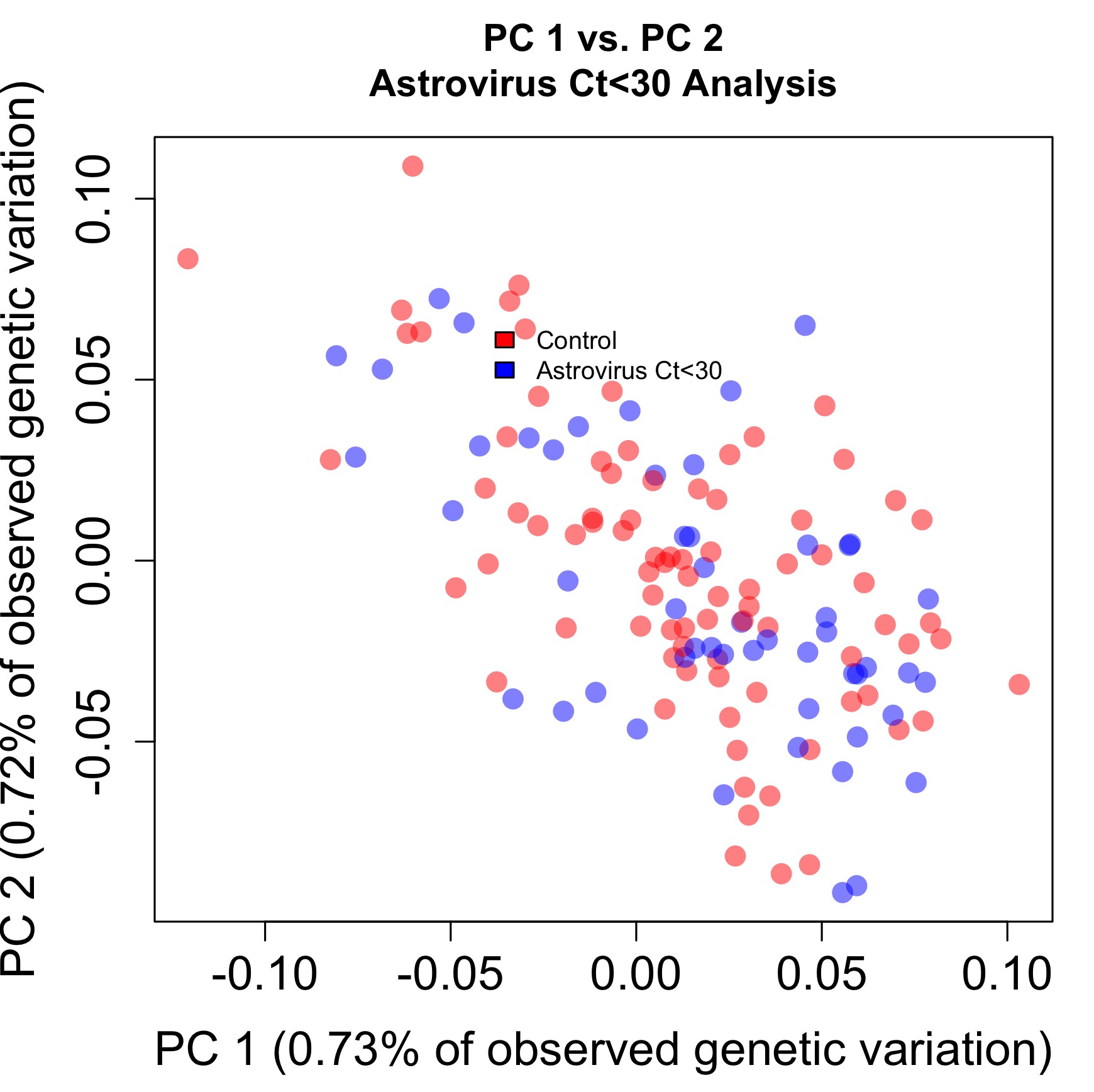
**

**Supplementary Figure 2.** Principal components analyses. **a)** PROVIDE: PC 1 vs. PC 2. **b)** CBC with outliers removed: PC 1 vs. PC 2.
