# Supplementary figures and images for: Genetic Susceptibility to Astrovirus Diarrhea in Bangladeshi Infants
Source: Open Forum Infect Dis. 2024 Mar 6;11(3):ofae045. doi: 10.1093/ofid/ofae045 (PMC10960603; doi:10.1093/ofid/ofae045)

**a)**

**
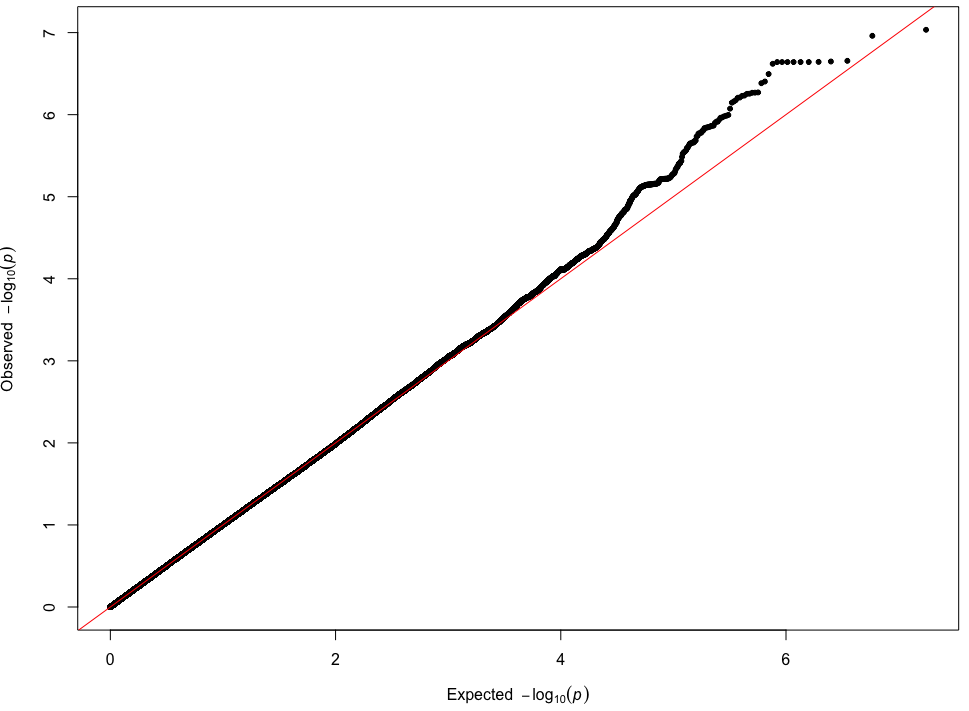
**

**b)**


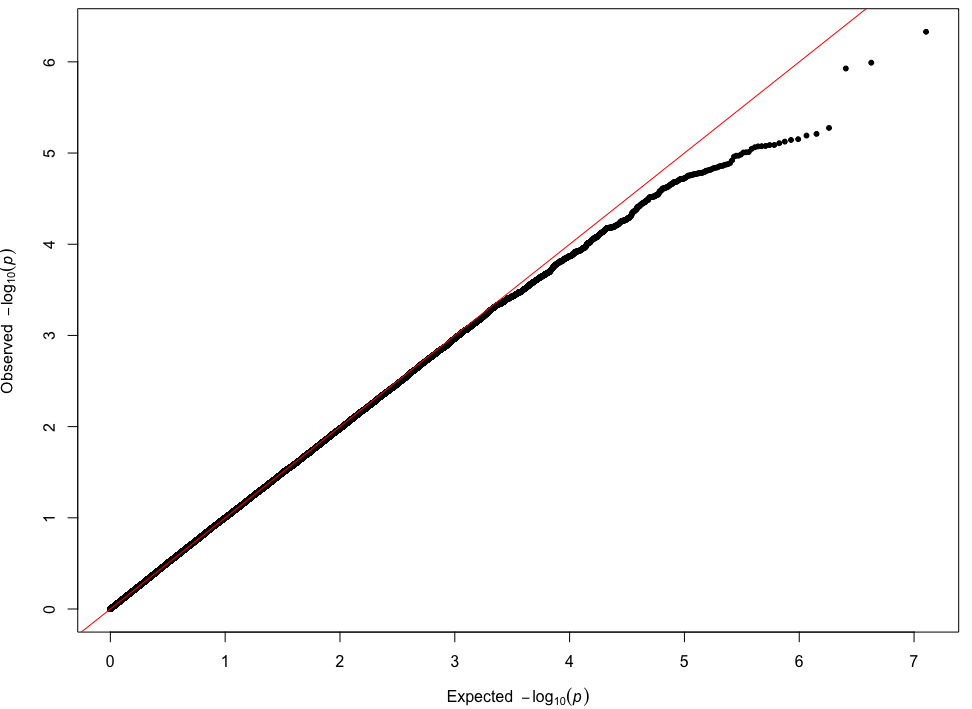


**Supplementary Figure 3.** Quantile-quantile plots of each cohort. **a)** PROVIDE, λ=1.003. **b)** CBC, λ=1.033.

Supplement: ofae045_Supplementary_Data [file ofae045_supplementary_data.zip › SuppFigure3.docx]
